# Longitudinal associations of plasma amino acid levels with recovery from malarial coma

**DOI:** 10.21203/rs.3.rs-4421190/v1

**Published:** 2024-05-24

**Authors:** Donald L. Granger, Daniel Ansong, Tsiri Agbenyega, Melinda S. Liddle, Benjamin A. Brinton, Devon C. Hale, Bert K. Lopansri, Richard Reithinger, Donal Bisanzio

**Affiliations:** a Division of Infectious Diseases, Department of Internal Medicine, University of Utah Spencer Fox Eccles School of Medicine, Salt Lake City, UT USA; b George H. Wahlen Veterans Affairs Medical Center, Salt Lake City, UT USA; c Department of Pediatrics, Komfo Anokye Teaching Hospital, Kumasi, Ghana; d Intermountain Health Care, Salt Lake City, UT USA; e Department of Psychiatry, North Shore University Hospital, Glen Oaks, NY USA; f International Development Group, Research Triangle Institute International, Washington, DC USA

**Keywords:** cerebral malaria, Blantyre coma score, amino acids, generalized linear mixed-effects model, tetrahydrobiopterin, glyceryl lipid ethers

## Abstract

**Background:**

Disordered amino acid metabolism is observed in cerebral malaria (CM). We sought to determine whether abnormal amino acid concentrations were associated with level of consciousness in children recovering from coma. We quantified 21 amino acids and coma scores longitudinally and analyzed data for associations.

**Methods:**

In a prospective observational study, we enrolled 42 children with CM. We measured amino acid levels at entry and at frequent intervals thereafter and assessed consciousness by Blantyre Coma Scores (BCS). Thirty-six healthy children served as controls for in-country normal amino acid ranges. We employed logistic regression using a generalized linear mixed-effects model to assess associations between out-of-range amino acid levels and BCS.

**Results:**

At entry 16/21 amino acid levels were out-of-range. Longitudinal analysis revealed 10/21 out-of-range amino acids were significantly associated with BCS. Elevated phenylalanine levels showed the highest association with low BCS. This finding held when out-of-normal-range data were analyzed at each sampling time.

**Discussion:**

We provide longitudinal data for associations between abnormal amino acid levels and recovery from CM. Of 10 amino acids significantly associated with BCS, we propose that elevated phenylalanine may be a surrogate for impaired clearance of ether lipid mediators of inflammation contributing to CM pathogenesis.

Of the complications associated with *Plasmodium falciparum* infection, unarousable coma, the hallmark of cerebral malaria (CM), may lead to long-term disability and death [1, 2]. Recent research aided by autopsy and imaging indicates that coma may be the result of generalized brain swelling due to accumulation of sequestered infected erythrocytes in the microvasculature [3–6]. Sequestration is the physical adherence of infected cells to activated endothelial cells within brain capillaries and venules. Microscopic, sequestered foci with patchy distribution restrict blood flow with downstream consequences including anoxia and acidosis. Leakage of intravascular fluid and, in some cases, erythrocytes into adjacent brain parenchyma through endothelial junctions adds to pathogenic events [7, 8].

In addition to the anatomical pathology in the brain, there are systemic abnormalities involving carbohydrate [9, 10] lipid [11] and amino acid [12, 13] metabolism. None are specific for CM—indeed, they are also seen in septic syndrome and other severe inflammatory diseases [14–16]. Metabolic causes of coma in CM have been sought, but no convincing pathogenic mechanism has been found. Nevertheless, these changes may contribute to coma in ways not yet recognized.

In our research on the role of nitric oxide in malaria pathogenesis, we noted abnormalities in the plasma levels of several amino acids in children presenting with CM [17, 18]. All abnormal amino acid levels were below the normal ranges, except for one, phenylalanine, which was consistently above 80 μM. Healthy controls rarely exceeded this cutoff concentration. With resolution of coma in treated survivors, phenylalanine levels normalized. We questioned whether longitudinal measurements of abnormal amino acid levels might be associated with the level of consciousness as children recovered. Such analysis may uncover an amino acid or group of amino acids involved in *P. falciparum* pathogenesis.

To address this question, we conducted a prospective observational study in Ghana, in which children entering hospital in coma due to malaria were observed frequently (every 12 – 24 hours) for 60 hours and their level of consciousness was quantified by Blantyre coma score(s) (BCS) [19]. At the same time intervals, blood samples were obtained and processed for 21 plasma amino acid levels. Our *a priori* hypothesis was that the levels of one or more amino acid(s) would be most closely associated with the BCS as participants regained consciousness. We enrolled a healthy control group to determine whether amino acid levels in Ghanaian children matched our reference laboratory normal ranges for healthy children in the US. The control group also provided disease versus normal comparisons at the time of presentation to hospital.

## METHODS

### Study design and site.

We conducted a prospective, observational, longitudinal study at Komfo Anokye Teaching Hospital in Kumasi, Ashanti Region, Ghana (6.697479°N 1.631690°W) from 2004 to 2006. We enrolled children who presented to the hospital with CM, as well as healthy control children who were asymptomatic hospital visitors or attendees at well-clinic check-ups. Children with uncomplicated malaria were not included, as our study was designed to address amino acid levels associated with recovery from coma. Study participants largely resided in Kumasi and its surrounding districts. The study was conducted by senior Ghanaian physicians in the Department of Pediatrics assisted by infectious diseases faculty, fellows, and medical students from the University of Utah. Plasma samples for amino acid analysis were obtained at enrollment (i.e., at admission) and at least every 24 hours during hospitalization.

### Enrollment criteria.

Children were 6 months to 6 years of age (median age, 2.8) WHO case definition for CM was used as inclusion criteria [20]: 1) any level of *Plasmodium falciparum* parasitemia on peripheral blood film; 2) unarousable coma as assessed by BCS ≤ 2 not attributable to hypoglycemia (i.e., blood glucose level < 40 mg/dl); 3) coma persisting more than 60 minutes after any convulsion; and 4) no other identifiable cause of coma. Exclusion criteria were any of the following: 1) microscopic or culture evidence of bacterial or viral co-infection; 2) oral or intravenous quinine or oral artemisinin combination therapy initiated > 18 hours prior to enrollment; 3) hemoglobin < 5 mg/dl when blood transfusion was unavailable at the study site.

Similar aged healthy children were prospectively enrolled as controls. Eligible children had no symptoms or signs of active illness, no febrile illness within the past 2 weeks, no history or evidence of an active inflammatory condition, and negative blood film for malaria parasites.

### Clinical evaluation and management.

Demographic information, clinical history, and physical examination were documented using standardized case report forms. Capillary blood samples were obtained for malaria thick and thin blood films and were prepared by Giemsa staining. Venous samples for routine laboratory analysis included complete blood count, electrolytes, creatinine, and lactate. Urine was obtained for dipstick analysis and culture. Blood and urine laboratory results were immediately available to clinicians. Blood cultures were obtained on all participants with CM. Lumbar puncture was done to investigate possible bacterial meningitis unless it was clear to the presiding clinicians that this diagnosis was unlikely. Thirty-four CM cases received lumbar puncture. Cerebrospinal fluid analysis included: a) determination of glucose and protein concentrations; b) cell count with differential performed by personnel trained in microscopy; c) Gram stain and bacterial and fungal cultures.

Children with CM received anti-malarial therapy and supportive care as per standard Ghanaian Ministry of Health protocols for the years during which the study was conducted (intravenous quinine or intravenous artesunate as recommended by WHO protocols). Treatment was initiated as soon as the diagnosis of malaria was suspected.

BCS was assessed at presentation and at least every 24 hours until hospital discharge or death. For some participants BCS was measured at admission and at 12-hour intervals for up to 60 hours. For each child at each interval, three clinicians independently assessed BCS. The assessments were done by a Ghanaian faculty physician, a US infectious diseases faculty or fellow, and a Ghanaian or US medical student. After assessment, the three clinicians met to obtain a consensus for BCS. For some patients this required returning to the bedside to review the findings. In all cases, consensus was reached by the three examiners and one BCS was recorded. At the time of BCS assessments the clinician examiners were unaware of any data pertaining to plasma amino acid concentrations.

### Plasma amino acid analysis.

Blood samples were collected into heparin tubes, mixed, and immediately centrifuged to sediment blood cells. Supernatant plasma was placed into polypropylene freezer tubes and stored at −80°C until shipment. Samples were transported in a liquid nitrogen dry shipper to the US for amino acid analysis. Amino acid analyses were performed at the Biochemical Genetics Section, ARUP Laboratories, University of Utah School of Medicine in collaboration with Dr. Marzia Pasquali. The amino acid analyzer employed ion exchange chromatography for separation and quantification. With two exceptions, all plasma amino acids were quantified. Exceptions were tryptophan, which emerged from the column lastly at a long retention time unsuitable for analysis, and hydroxyproline which gave frequent values of zero or below the level of sensitivity of our assay. Computer output results from the amino acid analyzer were electronically transferred to Excel spread sheets for subsequent data analysis.

### Statistical methods.

We compared data for CM cases and healthy controls at entry (Tables 1,2,3), which was performed with GraphPad Prism version 10.1.1. Data sets for each variable were tested for normality. Parametric data (Tables 1, 2) for both CM and healthy control groups were compared for significant difference using two-tailed Student’s t-test. For non-parametric data (Table 3) we used the two-tailed Mann-Whitney test. A significant difference was defined as P ≤ 0.05.

Association between BCS values and amino acid levels was investigated using statistical modeling. The aim of our model was to assess if an out-of-range level of each amino acid was linked to a low BCS. Since BCS is based on an ordinal discrete scale from 0 to 5, we employed an ordinal logistic regression, which is the most appropriate modeling technique for ordinal variables [21, 22]. The model was formulated using a generalized linear mixed-effects model (GLM-EM) [23]. It incorporated children as a random effect to account for variations in the sampling period. The ordinal logistic regression model describes the association between the dependent variable and independent variables by estimating odds ratios (OR). We used OR to identify the association between out-of-range amino acid levels and BCS. An OR significantly below 1.0 indicated that having an amino acid level outside the normal range was associated with low BCS. An OR above 1.0 indicated that having an amino acid level within the normal range was associated with a high BCS.

We also built a logistic regression GLM-EM to investigate the effect of time on the levels of amino acids [24]. This model aimed to identify the time threshold after which the levels of amino acids returned to the normal range. We considered an OR significantly above 1.0 as indicating a high probability that the amino acid level was normal at a given time, while an OR significantly below 1.0 indicated a high probability that the amino acid level was abnormal at a given time.

All statistical modeling was performed using BayesX software through R language interface [25, 26]. All P values were adjusted using the Bonferroni correction [27]. A significant association was defined as P ≤ 0.05.

### Ethics.

Our study was approved by the ethics committee at Komfo Anokye Teaching Hospital and the Institutional Review Board at the University of Utah, USA. Written informed consent was obtained from either parent or guardian of all participants. Consent forms were presented in Twi or in English, depending on the consenting parent or guardian preference. We followed US Department of Health and Human Services guidelines for human subjects research, the University of Utah guidelines, and the guidelines for the Komfo Anokye Teaching Hospital.

## RESULTS

### Clinical and laboratory findings.

We enrolled 42 children with CM due to *Plasmodium falciparum*; of CM cases, 6 were enrolled in 2004, 19 in 2005, and 17 in 2006. Thirty-six healthy controls were enrolled during these same years. The enrollment periods for each year were the same, i.e., during the long rainy season. Two CM participants were excluded from the analyses due to missing data; six children with CM died (14.3%) during hospitalization, all within the first 48 hours of admission.

Clinical characteristics for the two groups are listed in Table 1. The groups were closely matched for age, gender, and weight. Physical findings revealed the degree of illness in the CM group, including significant differences in pulse, respiratory rate, and temperature. Of those CM cases for whom clinical data was available, 68% experienced a witnessed seizure and 59% received diagnostic lumbar puncture. Historical data on most recent food intake indicated that plasma amino acid levels in CM participants were unlikely to be confounded by recent protein ingestion. The length of pre-admission symptoms (mean, 4 days) was consistent with the acute course of CM leading to coma prompting presentation at hospital.

Laboratory findings in CM cases were consistent with CM, including anemia, thrombocytopenia, and metabolic acidosis (Table 2). Elevated creatinine was likely due to acute kidney injury [28]. Hypoglycemia in CM participants was obscured by immediate intravenous glucose-containing fluid begun on all children presenting in coma.

### Plasma amino acid levels at entry.

Plasma samples collected from 40 CM enrollees at various time-points during hospitalization were available for amino acid analysis with one exception: a single sample at time zero. Of the 36 healthy control enrollees there were 6 samples unavailable for analysis because of insufficient plasma volume or missing samples.

For healthy controls, normality was usually the case. However, all amino acid data for the CM group showed non-parametric distributions. For 5 of the 21 amino acids (Asp, Cys, Leu, Tyr, Val), there were no significant differences between CM versus healthy controls at entry (Table 3). In all cases but one, the levels of the remaining amino acids (Ala, Arg, Cit, Glu, Gln, Gly, His, Ilu, Lys, Met, Orn, Pro, Ser, Tau, Thr) were significantly lower in the CM group compared to healthy controls. One amino acid (Phe) was significantly elevated in CM participants compared to healthy controls. Also shown in Table 3 are the normal ranges (mean ± 2SD) for each amino acid, which were established at the ARUP diagnostic laboratory based on a large age-dependent database of healthy US children. For Ghanaian healthy control children, amino acid levels were largely within the US normal ranges.

### Longitudinal association between BCS and amino acid levels outside the normal range.

Results obtained from our ordinal GLM-EM showed that BCS was significantly associated with amino acid levels outside the normal range for 10 of the 21 amino acids (Table 4). Nine of the 10 amino acids were associated with significant probability for having a low BCS, with a phenylalanine level outside the normal range being associated with the highest probability of having a low BCS. Conversely, valine out-of-range levels were associated with a high probability of having a high BCS.

Box plots showing out-of-range versus normal range individual samples for each amino acid at a given BCS are shown in Supplementary Data (Supplementary Figure 1, panels A – D).

### Logistic regression assessment of time to reach normal range levels for the 10 amino acids significantly associated with BCS.

**Figure 1.**
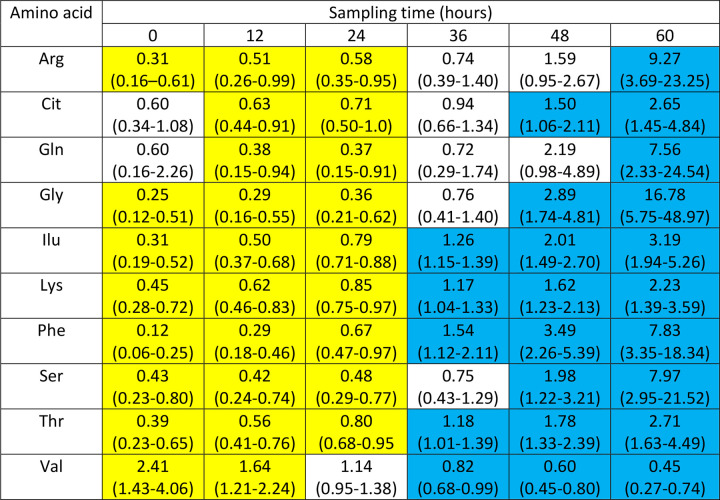
Effect of sampling time on having an amino acid level outside of, or within, the normal range. Results at each sampling time are OR (95% confidence intervals). Odd ratios in yellow cells highlight times significantly associated with an amino acid level outside the normal range; blue cells show times significantly associated with an amino acid level within the normal range; white cells show times with no significant association. Odds ratios less than one denote association with a level out-of-range, while odds ratios greater than one denote association with an amino acid level within the normal range.

**Figure 2.**
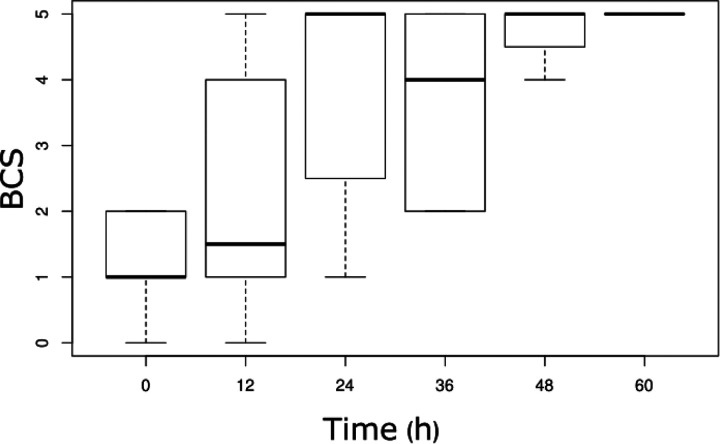
Box plot showing median coma scores with variance measures at sampling intervals. Boxes represent the interquartile range (IQR). Dark lines in boxes represent the median of the data at the times given. Vertical lines extending from the boxes indicate the minimum and maximum values within 1.5 times the IQR.

A logistic regression model was used to determine the time after which there was a high probability that amino acid levels reached the normal range. Almost all amino acids normalized by 48 – 60 hours post admission and initiation of treatment (Figure 1). However, our analyses showed marked variability across amino acids: for example, phenylalanine and isoleucine reached normal range at 36 hours, while for other amino acids it took 48 and 60 hours to reach the normal range. An outlier was valine, with significant normal range values at zero and 12 hours and abnormal range values at 36, 48 and 60 hours. The changes were associated with increasing BCS as time elapsed. For reference, Figure 2 shows BCS data at each sampling time.

## DISCUSSION

### Plasma amino acid abnormalities in CM at hospital entry

We defined abnormal amino acid levels based on age-dependent normal ranges for 21 amino acids as established for healthy USA children in our reference laboratory. To determine whether these normal ranges applied to healthy Ghanaian children, we enrolled age-matched healthy controls and measured their amino acid plasma levels. We found that healthy control levels were within normal ranges save for slight median decreases of cystine and glutamine (Table 3).

We compared amino acid levels in CM versus healthy control cohorts at hospital entry. Sixteen of the 21 amino acids were significantly different. Our results mirror those reported by others for children [13] and adults [12] with severe malaria, including CM. Fifteen of the 16 were below the normal range and one (phenylalanine) was elevated. The hypercatabolic state induced by the inflammatory response in severe malaria, in which amino acids are oxidatively degraded to yield chemical energy likely contributes to the low levels observed [29, 30] Additionally, gluconeogenesis in liver consumes glucogenic amino acids. Acute kidney injury associated with amino acid reabsorption abnormalities poses yet another source of loss [13]. Significantly low levels of glutamine, glutamate, proline, ornithine, citrulline and arginine comprise the pathway of *de novo* arginine synthesis [18] a finding consistent with low nitric oxide production [31]. Longitudinal data found low arginine levels associated with nitric oxide-dependent endothelial dysfunction [32].

### Longitudinal associations between out-of-range amino acid levels and BCS

Our results extended amino acid abnormalities by longitudinal measurements with analysis for association with BCS. By employing our GLM-EM model we found that ten of the sixteen out-of-range amino acids at entry were significantly associated with BCS (P ≤ 0.05, Table 4). Six of the ten (arginine, glycine, isoleucine, phenylalanine, threonine, and valine) showed high significance for association with BCS (P ≤ 0.01). Of this set, phenylalanine and valine stood out. Phenylalanine was the only amino acid with levels above the normal range in CM participants compared to healthy controls. Of all significant BCS-associated amino acids, phenylalanine showed the highest association (lowest odds ratio, 0.25). Valine out-or-range values associated with high BCS.

For the ten amino acids significantly associated with BCS we analyzed out-of-range values at each sampling time by calculating odds ratios with our GLM-EM model. With time, 9 of 10 amino acids normalized by 60 hours post admission and initiation of treatment (odds ratios > 1.0). At 24 to 36 hours post admission and initiation of treatment, levels transitioned to normal ranges for isoleucine, lysine, phenylalanine, and threonine. At these same time-points median BCS rose from about 1 to 4 – 5. Thus, regaining consciousness occurred at the time when these four amino acids normalized. The outlier was valine with significant normal range values at entry and at 12 hours with transition to abnormally low levels at 36, 48, and 60 hours post admission and initiation of treatment.

### Significance of association of abnormal valine levels with high BCS

Of the ten amino acids associated with BCS, we found a unique association between BCS and valine. Out-of-range valine levels bore significant probability to have high, rather than low, BCS. This finding could relate to the complex catabolism of the branched-chain amino acids (Leu, Ilu, Val). In experimental animal models and possibly in humans, high leucine levels antagonize valine degradation at the decarboxylation (keto acid dehydrogenase) step by competitive inhibition [33, 34]. This might delay valine catabolic kinetics resulting in out-of-range levels at later times during recovery, when BCS had risen to 4 or 5.

### Possible significance of hyperphenylalaninemia as a surrogate for malarial coma

Like other amino acids, the phenylalanine degradation supplies carbon to the TCA cycle for oxidation [35]. Why then did we and others find consistently elevated phenylalanine levels in CM? In a previous study we found a possible mechanism to explain hyperphenylalaninemia [36]. The liver enzyme, phenylalanine hydroxylase (PHA), requires the cofactor tetrahydrobiopterin (BH_4_) for mono-oxygenation of phenylalanine to yield its product, tyrosine [37]. In health biopterin is poised in the reduced state (BH_4_) such that with each phenylalanine to tyrosine reaction, the oxidized cofactors, biopterin (B) and dihydrobiopterin (BH_2_), are reduced back to BH_4_ via two pathways (recycling and salvage) [37]. The reducing equivalents for restoring biopterin to its reduced state are supplied by the nicotinamide adenine dinucleotides NADH and NADPH [37]. In the systemic compartment (i.e., plasma and liver) about 2/3 of biopterin is poised in the reduced state (BH_4_) [38]. This provides sufficient reducing power for tight regulation of plasma phenylalanine below 80 μM [39]. Phenylalanine regulation prevents hyperphenylalaninemia, which is toxic to the brain (e.g., in the congenital disease, phenylketonuria [39]). We found that, while total biopterins (B + BH2 + BH_4_) were slightly increased in CM, the percentage of reduced biopterin fell to 25% [36]. This suggests that hyperphenylalaninemia in CM results from a diminution of PAH catalysis in the liver due to insufficient BH_4_ availability, possibly a result of oxidative stress.

### Potential role of BH_4_ in CM pathogenesis

BH_4_ is a unique cofactor in that there are only 6 enzymes for which it is required [37, 40]. These are the enzymes for synthesis of catecholamines and serotonin in the brain, systemic and CNS synthesis of nitric oxide, PAH in liver, and a little studied liver enzyme for catabolism of membrane ether lipids, alkylglycerol mono-oxygenase (AGMO) [40–42]. Accumulation of membrane-derived ether lipids from impaired AGMO activity resulting from limited BH_4_ availability might adversely affect the CNS [43]. Blood-brain barrier permeability is markedly increased in experimental animals treated with short chain alkyl (ether) glycerols [44, 45]. Limiting AGMO activity could enhance circulating ether lipid mediators such as platelet activation factor (PAF). This possibility is yet to be investigated [46, 47]. There is evidence from malaria models that PAF could enhance sequestration of infected red cells if regulation control mechanisms fail [48, 49]. The possible role for an ether lipid(s) in malaria pathogenesis should be explored.

### Limitations

The cohort of 40 CM cases is small. BCS are subjective, therefore open to observer bias. Our linear mixed effects model can only assign association between out-of-range amino acid levels and BCS, and thus cannot establish cause and effect. Diagnosis of CM carries a significant false positive fraction [50]. However, 59% of our CM cohort received lumbar puncture to rule out meningitis, thus diminishing the likelihood of non-malarial causes of coma.

### Conclusion

Our longitudinal data on normalization of amino acid levels with recovery from malarial coma found a highly significant association for phenylalanine. Unlike other coma-associated amino acids, abnormal phenylalanine levels were above, not below, normal range. Hyperphenylalaninemia likely results from insufficient reducing power normally provided by tetrahydrobiopterin. Previously we found in CM cases, a marked diminution of tetrahydrobiopterin. We posit that the single other liver tetrahydrobiopterin-dependent enzyme, responsible for degrading biologically active ether lipids (AGMO), may be impaired. Elevated bioactive ether lipids are known to permeabilize the CNS blood-brain barrier and lead to platelet activation, events shown to be critical in CM pathogenesis. If impaired, restoration of AGMO activity could provide efficacious adjunctive therapy.

## Supplementary Material

1

## Figures and Tables

**Table 1.** Clinical findings at enrollment

	Study Group				
Variable	N^a^	Cerebral malaria^b^	N^a^	Healthy control^b^	P value^c^
Age (years)	40	2.8 (1.6 – 4.0)	36	3.0 (2.0 – 4.2)	0.3
Gender (% female)	40	63	36	56	0.6
Body Weight (Kg)	25	12 (11 – 13)	5	14 (9 – 16)	0.6
Pulse (beats per min)^d^	40	142 (135 – 148)	9	121 (103 – 138)	0.008
Mean blood pressure (mm Hg^0^)	25	78 (74 – 89)	9	91 (82 – 95)	0.07
Respirations (breaths per min)^e^	40	40 (32 – 48)	9	30 (24 – 32)	0.009
Temperature (°C)^d^	40	38.0 (37.6 – 38.4)	35	36.4 (36.2 – 36.6)	<0.0001
Last food taken (hours)	32	18.8 (12.2 – 25.3)	-	ND^f^	-
Last liquid taken (hours)	31	14.2 (10.8 – 17.7)	-	ND^f^	-
Pre-admission symptoms (days)	39	3.8(3.0 – 4.6)	-	NA^g^	-
Witnessed seizure No. (%)^h^	34	23 (68)	-	NA^g^	-
Lumbar puncture No. (%)^i^	34	20 (59)	-	NA^g^	-

a Number of participants evaluable from source data.

b Values are medians (IQR) unless otherwise noted.

c Mann-Whitney test unless otherwise noted. Significant difference defined as *p* ≤ 0.05.

d Unpaired t-test. Values are means (95% CI)

e 53% of cerebral malaria cases exhibited Kussmaul-Kien respirations (air hunger)

f Not determined

g Not applicable

h Observed by physician during hospitalization.

I Lumbar puncture omitted in some cases based on clinical decision as deemed unnecessary. Three CSF samples were contaminated with nonpathogenic bacterial growth. No CSF samples were consistent with bacterial/fungal meningitis based on cell count, Gram stain and culture.

**Table 2.** Laboratory results on admission to hospital

	Study group				
Laboratory test	N^a^	Cerebral malaria^b^	N^a^	Healthy control^a^	P value^c^
Hemoglobin conc. (mg/dl)	40	6.9 (6.3 – 7.5)	35	11.0 (10.5 – 11.4)	<0.0001
White blood cell count (x10^3^/μl)	40	12.3 (10.0 – 14.7)	35	10.5 (9.2 – 11.7)	0.18
Platelet count (x10^3^/μl)	40	64.2 (46.7 – 81.7)	35	252 (201 – 303)	<0.0001
Glucose conc. (mM)^d^	32	10.8 (7.7 – 13.9)	29	5.0 (4.5 – 5.6)	0.0007
Creatinine conc. (mM)^e^	19	73.8 (62.6 – 85.0)	21	59.0 (55.1 – 62.9)	0.01
Lactate conc. (mM)	26	4.55 (3.49 – 5.61)	25	1.81 (1.57 – 2.06)	<0.0001
Parasitemia (trophs x10^3^/μl)^f^	40	64.4 (43.6 – 95.1)	36	0	NA^g^

a Number of participants

b Values are medians (IQR) unless noted otherwise.

c Mann-Whitney test for significance (P ≤ 0.05) unless noted.

d High glucose values in majority of cerebral malaria patients explained by blood sample taken during infusion of glucose-containing iv fluids.

e Values are means (95%CI). Unpaired t-test for significance (P ≤ 0.05).

f Geometric mean (95% confidence limits of geometric mean)

g Not Applicable

**Table 3.** Plasma amino acid levels at entry in children with cerebral malaria versus healthy controls.

		Plasma amino acid concentration^c^		
Amino acid^a^	Normal range (μmoles/L)^b^	Cerebral malaria	Healthy control	P value^d^
Alanine	240 – 600	250 (151 – 341)	433 (383 – 470)	<0.0001
Arginine	40 – 160	28 (23 – 38)	63 (53 – 96)	<0.0001
Aspartate	0 – 20	10 (7 – 15)	11 (9 – 18)	NS
Citrulline	10 – 60	7 (4 – 10)	25 (17 – 32)	<0.0001
Cystine	7 – 70	5 (3 – 13)	6 (2 – 9)	NS
Glutamate	10 – 120	60 (40 – 89)	121 (100 – 152)	<0.0001
Glutamine	410 – 700	248 (201 – 312)	402 (303 – 444)	<0.0001
Glycine	140 – 490	120 (100 – 137)	248 (216 – 342)	<0.0001
Histidine	50 – 130	66 (53 – 99)	94 (74 – 119)	0.0007
Isoleucine	30 – 130	25 (19 – 49)	62 (44 – 76)	<0.0001
Leucine	60 – 230	116 (90 – 157)	120 (85 – 142)	NS
Lysine	80 – 250	75 (61 – 95)	119 (98 – 145)	<0.0001
Methionine	17 – 53	15 (10 – 20)	23 (18 – 29)	<0.0001
Ornithine	20 – 135	24 (16 – 41)	77 (66 – 100)	<0.0001
Phenylalanine	30 – 80	111 (76 – 157)	66 (57 – 79)	<0.0001
Proline	110 – 500	153 (86 – 233)	254 (173 – 308)	0.0005
Serine	60 – 200	59 (51 – 75)	143 (131 – 169)	<0.0001
Taurine	25 – 80	53 (35 – 78)	91 (75 – 118)	<0.0001
Threonine	60 – 220	49 (43 – 58)	90 (70 109)	<0.0001
Tyrosine	30 – 120	57 (40 – 82)	70 (60 – 83)	NS
Valine	140 – 350	198 (150 – 270)	192 (148 – 224)	NS

a Results for hydroxyproline were below the level of detection for our chromatography platform and hence the data set for this amino acid is excluded from analysis. The ion exchange chromatography method used did not include L-tryptophan.

b Range of normal clinical values (μM/L) for the age group of our CM and healthy control cohorts. Normal ranges established for US children at ARUP Laboratories, Salt Lake City, UT., USA.

c Values are median [IQR] in μM/L. Cerebral malaria N = 40; Healthy Control N = 30.

d P value > 0.05 considered not significant (NS).

**Table 4:** Association between BCS and amino acid level outside the normal range^1^

Amino Acid^2^	OR (95% CI)
Alanine	1.29 (0.84; 1.99)
Arginine**	0.47 (0.31; 0.71)
Aspartate	1.47 (0.76; 2.85)
Citrulline*	0.65 (0.41; 1.02)
Cystine	0.66 (0.39; 1.13)
Glutamate	0.96 (1.96; 0.47)
Glutamine*	0.45 (0.20; 0.97)
Glycine**	0.34 (0.22; 0.53)
Histidine	1.28 (0.80; 2.05)
Isoleucine**	0.57 (0.37; 0.88)
Leucine	1.30 (0.70; 2.43)
Lysine*	0.66 (0.42; 1.01)
Methionine	0.74 (0.49; 1.11)
Ornithine	0.71 (0.45; 1.10)
Phenylalanine**	0.25 (0.16; 0.40)
Proline	0.71 (0.46; 1.09)
Serine*	0.60 (0.39; 0.94)
Taurine	0.73 (0.47; 1.14)
Threonine**	0.51 (0.32; 0.82)
Tyrosine	1.28 (0.68; 2.42)
Valine**	2.48 (1.58; 3.89)

1 The table reports the odds ratio (OR) estimates obtained from the ordinal regression model.

Abbreviation: CI, confidence interval.

2 *P < 0.05; ** P < 0.01.

## References

[1] Marsh K, Forster D, Waruiru C, Indicators of life-threatening malaria in African children. N Engl J Med 1995; 332:1399–404. 7723795 10.1056/NEJM199505253322102

[2] Anonymous. World Malaria Report 2021. Geneva: World Health Organization; 2021., 2021.

[3] Seydel KB, Kampondeni SD, Valim C, Brain swelling and death in children with cerebral malaria. N Engl J Med 2015; 372:1126–37. 25785970 10.1056/NEJMoa1400116 PMC4450675

[4] Potchen MJ, Kampondeni SD, Seydel KB, 1.5 Tesla Magnetic Resonance Imaging to Investigate Potential Etiologies of Brain Swelling in Pediatric Cerebral Malaria. Am J Trop Med Hyg 2018; 98:497–504. 29313473 10.4269/ajtmh.17-0309 PMC5929182

[5] Kampondeni S, Seydel KB, Zhang B, Amount of Brain Edema Correlates With Neurologic Recovery in Pediatric Cerebral Malaria. Pediatr Infect Dis J 2020; 39:277–82. 32168246 10.1097/INF.0000000000002573

[6] Poespoprodjo JR, Douglas NM, Ansong D, Kho S, Anstey NM. Malaria. Lancet 2023; 402:2328–45. 37924827 10.1016/S0140-6736(23)01249-7

[7] Darling TK, Mimche PN, Bray C, EphA2 contributes to disruption of the blood-brain barrier in cerebral malaria. PLoS Pathog 2020; 16:e1008261. 31999807 10.1371/journal.ppat.1008261 PMC6991964

[8] Moxon C, Alhamdi Y, Storm J, Parasite histones mediate blood-brain barrier disruption in cerebral malaria. Clin Med (Lond) 2020; 20:s96–s7. 32409404 10.7861/clinmed.20-2-s96 PMC7243569

[9] White NJ, Warrell DA, Chanthavanich P, Severe hypoglycemia and hyperinsulinemia in falciparum malaria. N Engl J Med 1983; 309:61–6. 6343877 10.1056/NEJM198307143090201

[10] Planche T, Krishna S. Severe malaria: metabolic complications. Curr Mol Med 2006; 6:141–53. 16515507 10.2174/156652406776055177

[11] Herdman MT, Sriboonvorakul N, Leopold SJ, The role of previously unmeasured organic acids in the pathogenesis of severe malaria. Crit Care 2015; 19:317. 26343146 10.1186/s13054-015-1023-5 PMC4561438

[12] Leopold SJ, Apinan S, Ghose A, Amino acid derangements in adults with severe falciparum malaria. Sci Rep 2019; 9:6602. 31036854 10.1038/s41598-019-43044-6 PMC6488658

[13] Conroy AL, Tran TM, Bond C, Plasma Amino Acid Concentrations in Children With Severe Malaria Are Associated With Mortality and Worse Long-term Kidney and Cognitive Outcomes. The Journal of Infectious Diseases 2022; 226:2215–25. 36179241 10.1093/infdis/jiac392 PMC10205609

[14] Dinarello CA. Interleukin-1 and the pathogenesis of the acute-phase response. N Engl J Med 1984; 311:1413–8. 6208485 10.1056/NEJM198411293112205

[15] Lundblad RL. Biotechnology of plasma proteins. 2012 Biotechnology of Plasma Proteins).

[16] van Gassel RJJ, Baggerman MR, van de Poll MCG. Metabolic aspects of muscle wasting during critical illness. Curr Opin Clin Nutr Metab Care 2020; 23:96–101. 31904602 10.1097/MCO.0000000000000628 PMC7015189

[17] Lopansri BK, Anstey NM, Stoddard GJ, Elevated plasma phenylalanine in severe malaria and implications for pathophysiology of neurological complications. Infect Immun 2006; 74:3355–9. 16714564 10.1128/IAI.02106-05 PMC1479261

[18] Rubach MP, Zhang H, Florence SM, Kinetic and Cross-Sectional Studies on the Genesis of Hypoargininemia in Severe Pediatric Plasmodium falciparum Malaria. Infect Immun 2019; 87. 10.1128/IAI.00655-18 PMC6434111 30718287

[19] Molyneux ME, Taylor TE, Wirima JJ, Borgstein A. Clinical features and prognostic indicators in paediatric cerebral malaria: a study of 131 comatose Malawian children. Q J Med 1989; 71:441–59. 2690177

[20] WHO. Severe falciparum malaria. Trans R Soc Trop Med Hyg 2000; 94.

[21] Armstrong BG, Sloan M. Ordinal regression models for epidemiologic data. Am J Epidemiol 1989; 129:191–204. 2910061 10.1093/oxfordjournals.aje.a115109

[22] Norris CM, Ghali WA, Saunders LD, Ordinal regression model and the linear regression model were superior to the logistic regression models. J Clin Epidemiol 2006; 59:448–56. 16632132 10.1016/j.jclinepi.2005.09.007

[23] Hedeker D. A mixed-effects multinomial logistic regression model. Stat Med 2003; 22:1433–46. 12704607 10.1002/sim.1522

[24] Ugwu CLJ, Zewotir TT. Using mixed effects logistic regression models for complex survey data on malaria rapid diagnostic test results. Malar J 2018; 17:453. 30518399 10.1186/s12936-018-2604-y PMC6282337

[25] Umlauf N, Adler D, Kneib T, Lang S, Zeileis A. Structured additive regression models: An R interface to BayesX. 2015. Journal of Statistical Software 63:1–46.

[26] Team RC. R: a language and environment for statistical computing. Vienna: R Foundation for Statistical Computing. (No Title) 2021.

[27] Cabin RJ, Mitchell RJ. To Bonferroni or not to Bonferroni: when and how are the questions. Bulletin of the ecological society of America 2000; 81:246–8.

[28] Batte A, Berrens Z, Murphy K, Malaria-Associated Acute Kidney Injury in African Children: Prevalence, Pathophysiology, Impact, and Management Challenges. Int J Nephrol Renovasc Dis 2021; 14:235–53. 34267538 10.2147/IJNRD.S239157 PMC8276826

[29] Basler T, Meier-Hellmann A, Bredle D, Reinhart K. Amino acid imbalance early in septic encephalopathy. Intensive Care Med 2002; 28:293–8. 11904658 10.1007/s00134-002-1217-6

[30] Bröer S, Bröer A. Amino acid homeostasis and signalling in mammalian cells and organisms. Biochem J 2017; 474:1935–63. 28546457 10.1042/BCJ20160822 PMC5444488

[31] Anstey NM, Weinberg JB, Hassanali MY, Nitric oxide in Tanzanian children with malaria: inverse relationship between malaria severity and nitric oxide production/nitric oxide synthase type 2 expression. J Exp Med 1996; 184:557–67. 8760809 10.1084/jem.184.2.557 PMC2192721

[32] Yeo TW, Lampah DA, Gitawati R, Recovery of endothelial function in severe falciparum malaria: relationship with improvement in plasma L-arginine and blood lactate concentrations. J Infect Dis 2008; 198:602–8. 18605903 10.1086/590209 PMC2709993

[33] Harper AE, Miller RH, Block KP. Branched-chain amino acid metabolism. Annu Rev Nutr 1984; 4:409–54. 6380539 10.1146/annurev.nu.04.070184.002205

[34] Staten MA, Bier DM, Matthews DE. Regulation of valine metabolism in man: a stable isotope study. Am J Clin Nutr 1984; 40:1224–34. 6439027 10.1093/ajcn/40.6.1224

[35] Castillo L, Yu YM, Marchini JS, Phenylalanine and tyrosine kinetics in critically ill children with sepsis. Pediatr Res 1994; 35:580–8. 8065841

[36] Rubach MP, Mukemba J, Florence S, Impaired systemic tetrahydrobiopterin bioavailability and increased oxidized biopterins in pediatric falciparum malaria: association with disease severity. PLoS Pathog 2015; 11:e1004655. 25764173 10.1371/journal.ppat.1004655 PMC4357384

[37] Blau N TB, Cotton RGH, Hyland K. Disorders of tetrahydrobiopterin and related biogenic amines. 8th ed. Vol. II. New York, New York: McGraw-Hill, 2001.

[38] Hyland K. Estimation of tetrahydro, dihydro and fully oxidised pterins by high-performance liquid chromatography using sequential electrochemical and fluorometric detection. J Chromatogr 1985; 343:35–41. 4066860 10.1016/s0378-4347(00)84565-x

[39] Kaufman CSaS. Hyperphenylalaninemia: Phenylalanine hydroxylase deficiency. In: Scriver AB CR, Sly WS, Valle D, ed. The Metabolic and Molecular Bases of Inherited Disease. 8th Edition ed. Vol. II. New York, NY: McGraw-Hill, 2001:1667–724.

[40] Werner ER. Three classes of tetrahydrobiopterin-dependent enzymes. Pteridines 2013; 24:7–11.

[41] Watschinger K, Werner ER. Alkylglycerol monooxygenase. IUBMB Life 2013; 65:366–72. 23441072 10.1002/iub.1143 PMC3617469

[42] Sailer S, Keller MA, Werner ER, Watschinger K. The Emerging Physiological Role of AGMO 10 Years after Its Gene Identification. Life (Basel) 2021; 11. 10.3390/life11020088 PMC7911779 33530536

[43] Dorninger F, Forss-Petter S, Wimmer I, Berger J. Plasmalogens, platelet-activating factor and beyond - Ether lipids in signaling and neurodegeneration. Neurobiol Dis 2020; 145:105061. 32861763 10.1016/j.nbd.2020.105061 PMC7116601

[44] Erdlenbruch B, Alipour M, Fricker G, Alkylglycerol opening of the blood-brain barrier to small and large fluorescence markers in normal and C6 glioma-bearing rats and isolated rat brain capillaries. Br J Pharmacol 2003; 140:1201–10. 14597599 10.1038/sj.bjp.0705554 PMC1574140

[45] Erdlenbruch B, Jendrossek V, Eibl H, Lakomek M. Transient and controllable opening of the blood-brain barrier to cytostatic and antibiotic agents by alkylglycerols in rats. Exp Brain Res 2000; 135:417–22. 11146820 10.1007/s002210000553

[46] Stafforini DM, McIntyre TM, Zimmerman GA, Prescott SM. Platelet-activating factor, a pleiotrophic mediator of physiological and pathological processes. Crit Rev Clin Lab Sci 2003; 40:643–72. 14708958 10.1080/714037693

[47] Gupta S, Seydel K, Miranda-Roman MA, Extensive alterations of blood metabolites in pediatric cerebral malaria. PLoS One 2017; 12:e0175686. 28426698 10.1371/journal.pone.0175686 PMC5398544

[48] Lacerda-Queiroz N, Rodrigues DH, Vilela MC, Platelet-activating factor receptor is essential for the development of experimental cerebral malaria. Am J Pathol 2012; 180:246–55. 22079430 10.1016/j.ajpath.2011.09.038

[49] Lacerda-Queiroz N, Rachid MA, Teixeira MM, Teixeira AL. The role of platelet-activating factor receptor (PAFR) in lung pathology during experimental malaria. Int J Parasitol 2013; 43:11–5. 23260771 10.1016/j.ijpara.2012.11.008

[50] Taylor TE, Fu WJ, Carr RA, Differentiating the pathologies of cerebral malaria by postmortem parasite counts. Nat Med 2004; 10:143–5. 14745442 10.1038/nm986

