# Supplementary material for: Longitudinal associations of plasma amino acid levels with recovery from malarial coma
Source: Res Sq. 2024 May 24:rs.3.rs-4421190. Preprint. [Version 1] doi: 10.21203/rs.3.rs-4421190/v1 (PMC11142354; doi:10.21203/rs.3.rs-4421190/v1)
Supplement: 1 [file NIHPPrs4421190V1-supplement-1.pdf]

SUPPLEMENTARY DATA

Supplementary Figure 1. Box plots show out-of-range versus normal-range amino acid plasma levels at given BCS.

Figure 1A – 1D show results for all 21 amino acids analyzed. Colored circles represent individual amino acid levels for participants with cerebral malaria. An Asterix denotes significant ($P < 0.05$) association between amino acid levels within (yellow circles) or outside (red circles) the normal ranges at given Blantyre Coma Scores as determined by our generalized linear mixed-effects model (GLM-EM).

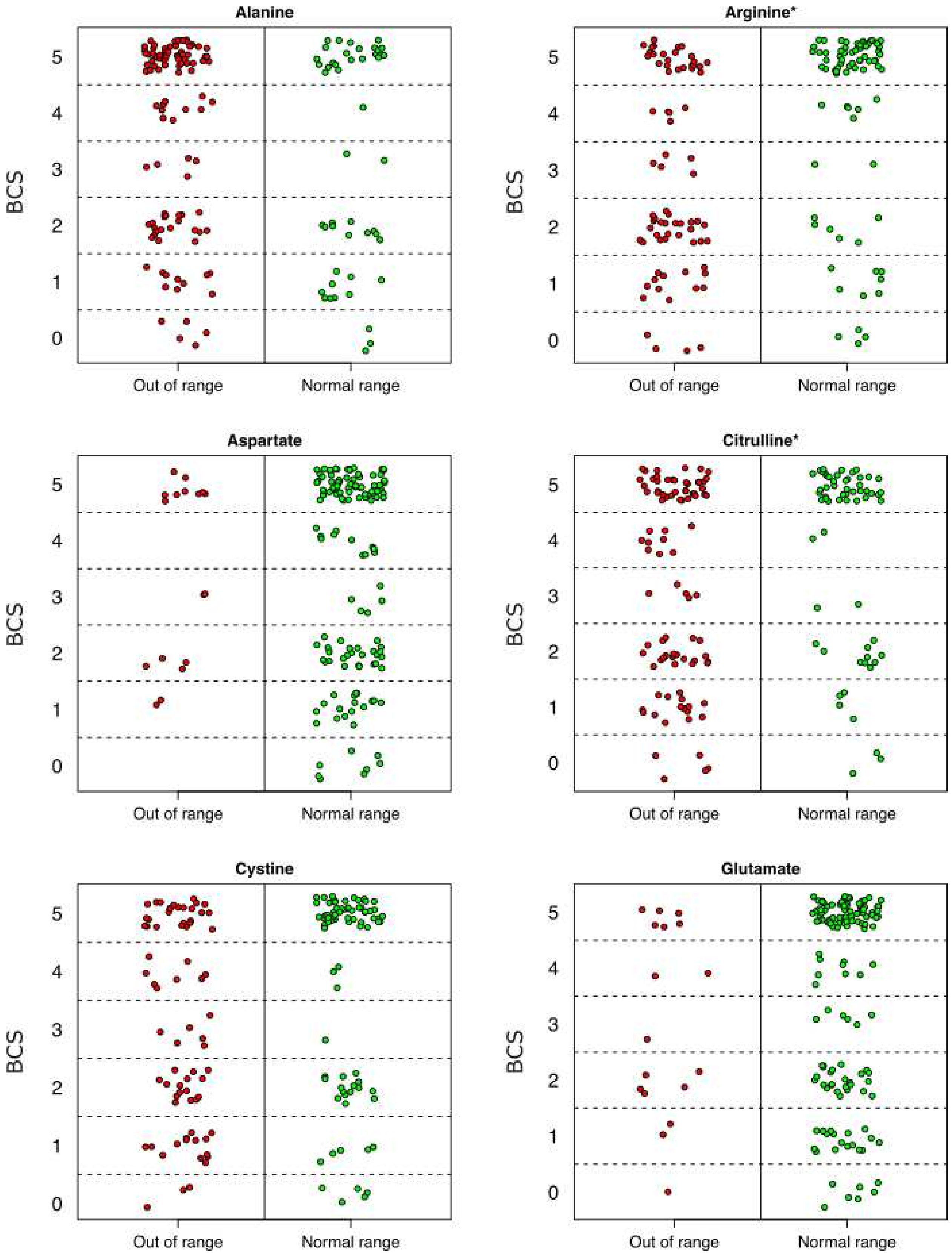
Alanine
Arginine*
Aspartate
Citrulline*
Cystine
Glutamate
BCS
0
1
2
3
4
5
Out of range
Normal range

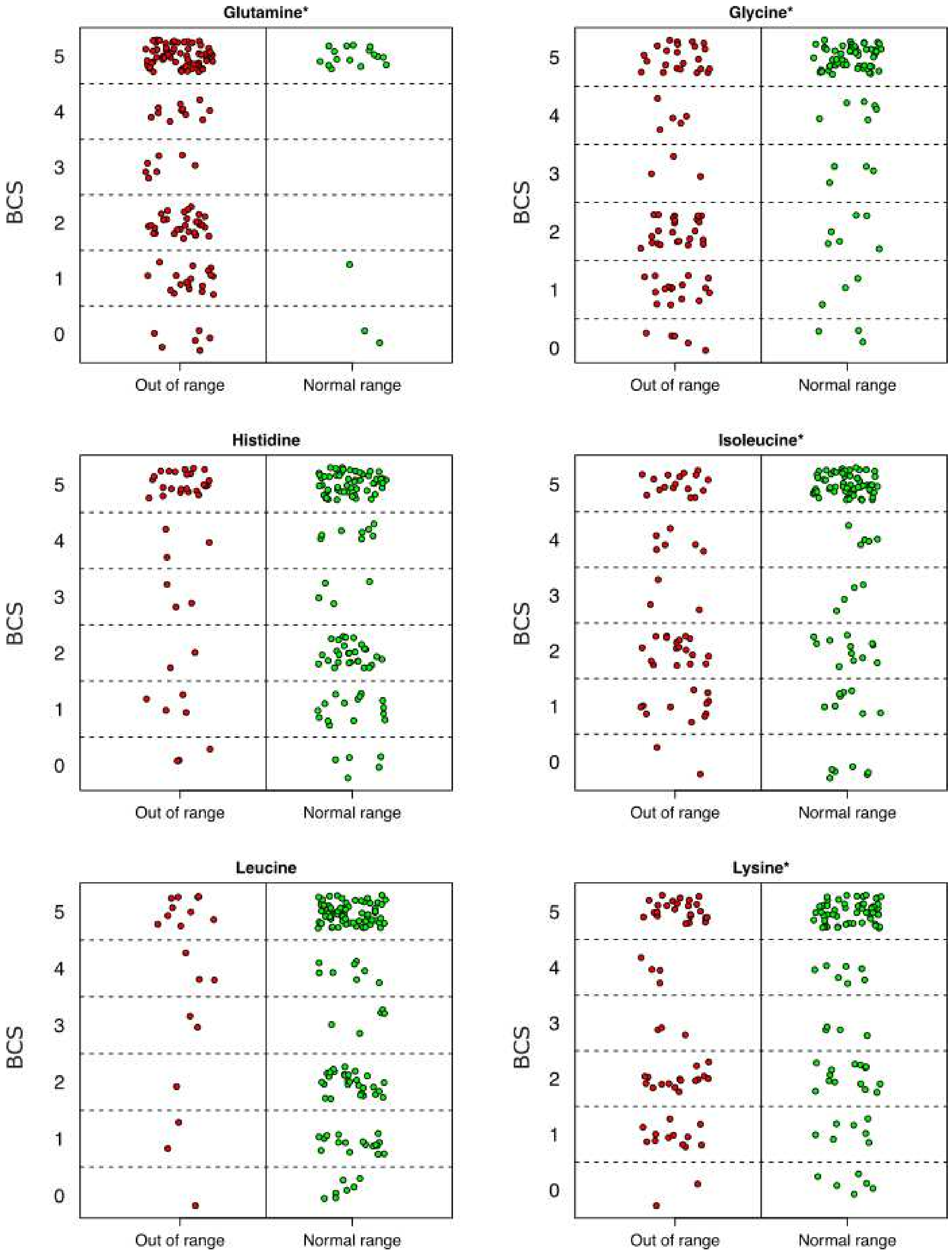
Glutamine*
Glycine*
Histidine
Isoleucine*
Leucine
Lysine*
BCS
0
1
2
3
4
5
Out of range
Normal range

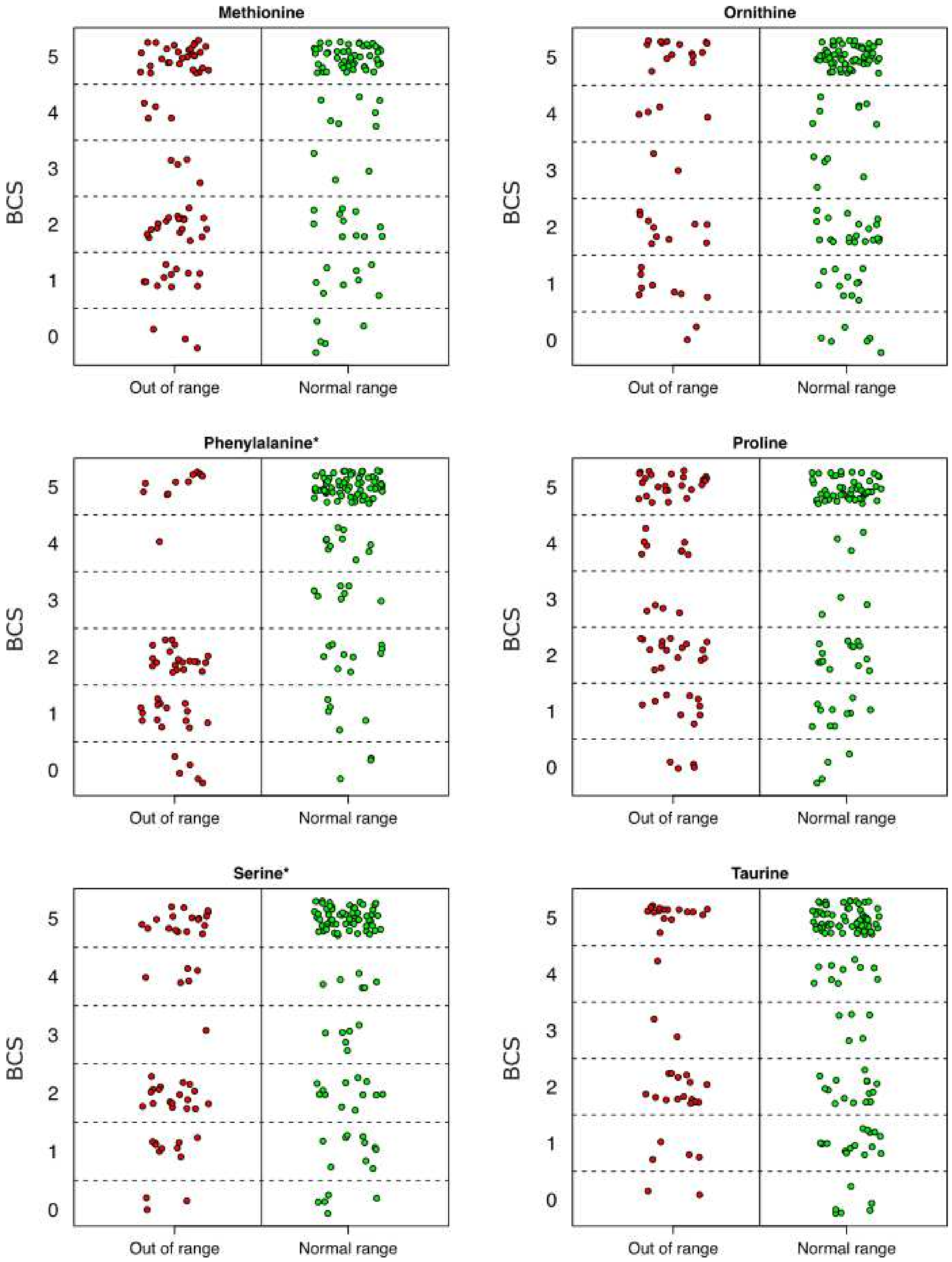
Methionine
Ornithine
Phenylalanine*
Proline
Serine*
Taurine
BCS
0
1
2
3
4
5
Out of range
Normal range

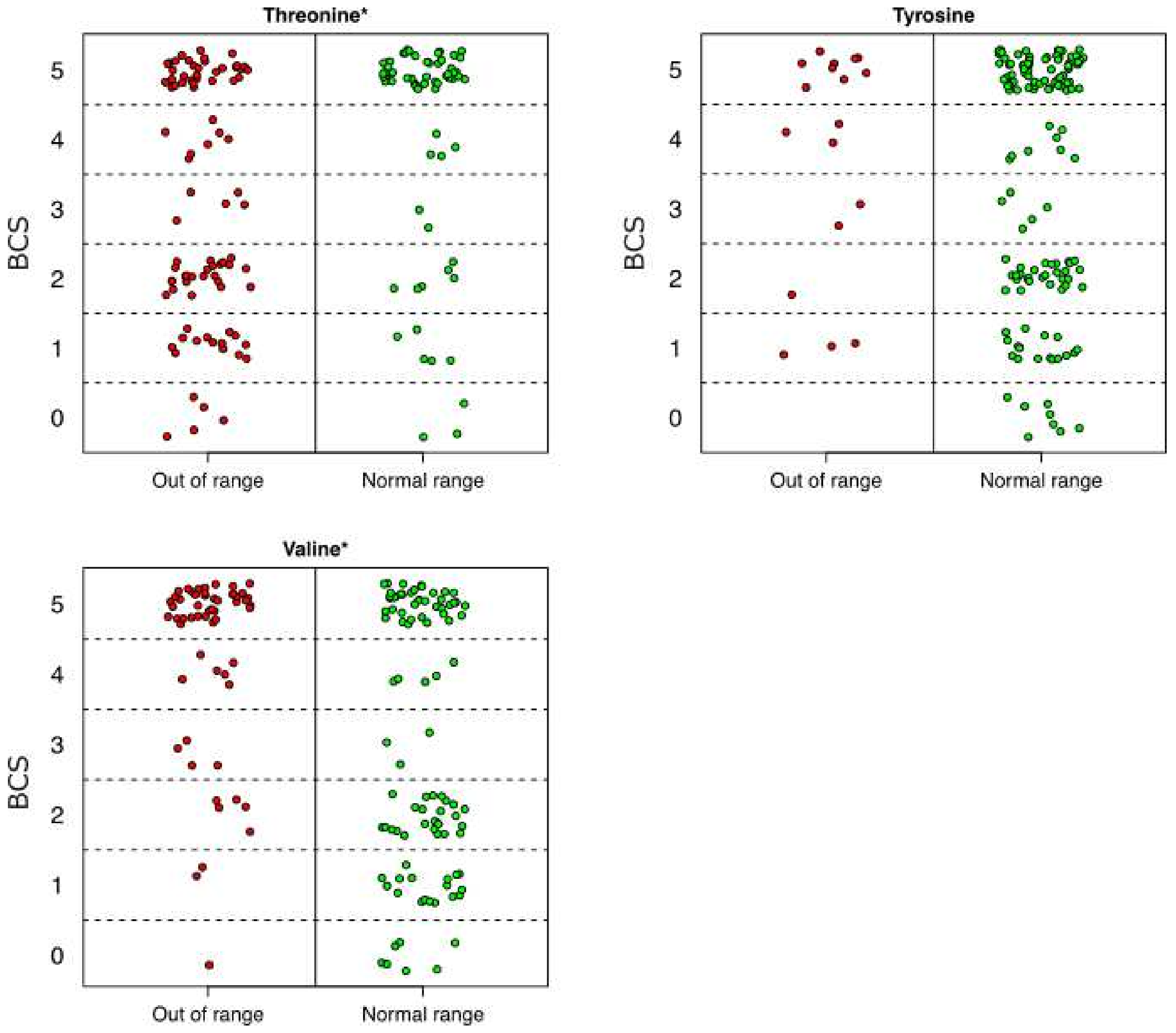
Threonine*
Tyrosine
Valine*
BCS
5
4
3
2
1
0
Out of range
Normal range
